# Tumor immune microenvironment in endometrial cancer of different molecular subtypes: evidence from a retrospective observational study

**DOI:** 10.3389/fimmu.2022.1035616

**Published:** 2022-11-24

**Authors:** Yibo Dai, Luyang Zhao, Dingchao Hua, Lina Cui, Xiaobo Zhang, Nan Kang, Linlin Qu, Liwei Li, He Li, Danhua Shen, Zhiqi Wang, Jianliu Wang

**Affiliations:** ^1^Department of Obstetrics and Gynecology, Peking University People’s Hospital, Beijing, China; ^2^Department of Medical Affairs, 3D Medicines Inc., Shanghai, China; ^3^Department of Pathology, Peking University People’s Hospital, Beijing, China

**Keywords:** uterine neoplasms (MeSH), molecular subtype, tumor immune microenvironment, prognosis, immunotherapy

## Abstract

**Objective:**

Tumor immune microenvironmental features may predict survival and guide treatment. This study aimed to comprehensively decipher the immunological features of different molecular subtypes of endometrial cancer.

**Methods:**

In this retrospective study, 26 patients with primary endometrial cancer and four with recurrent disease treated in our center from December 2018 to November 2021 were included. Next-generation sequencing was performed on tumor samples. Patients were classified into four subtypes, including *POLE* mutant, microsatellite instability high (MSI-H), no specific molecular profile (NSMP) and *TP53* mutant subtypes. Tumor-infiltrating immune cells were quantified using multiplex immunofluorescence assays.

**Results:**

Of the 26 primary endometrial cancer cases, three were *POLE* mutant, six were MSI-H, eight were NSMP and nine were *TP53* mutant. Of the four recurrent cases, two belonged to the NSMP subtype and two belonged to the *TP53* mutant subtype. The tumor mutation burden (TMB) levels of *POLE* mutant and MSI-H cases were significantly higher than that of the other two subtypes (*p<* 0.001). We combined *POLE* mutant and MSI-H subtypes into the TMB high (TMB-H) subtype. The TMB-H subtype showed a high degree of infiltration of CD8^+^ T cells. In the NSMP subtype, the overall degree of intra-tumoral infiltrating immune cells was low. In the *TP53* mutant subtype, the densities of both PD-L1^+^ macrophages (*p* = 0.047) and PD-1^+^ T cells (*p* = 0.034) in tumor parenchyma were the highest among the four subtypes.

**Conclusion:**

Endometrial cancer of TMB-H, NSMP and *TP53* mutant subtypes displayed phenotypes of normal immune response, absence of immune infiltration, and suppressed immune response, respectively. These features may provide mechanistic explanations for the differences in patients’ prognosis and efficacy of immune checkpoint blockade therapies among different endometrial cancer subtypes.

## Introduction

In the past decade, the development of high-throughput sequencing technologies and computational algorithms has facilitated the understanding of cancer genomics. In endometrial cancer (EC), the establishment of molecular subtypes by the Cancer Genome Atlas (TCGA) consortium (1), on the one hand, has affected patient stratification, promoting individualized clinical management. In 2021, the National Comprehensive Cancer Network (NCCN) guidelines for uterine neoplasms recommended molecular subtyping in EC diagnosis (2). In addition, the European Society of Gynecologic Oncology (ESGO)/European Society for Radiotherapy and Oncology (ESTRO)/European Society of Pathology (ESP) guideline for EC further incorporated molecular subtypes into the risk stratification system for guiding postoperative adjuvant therapies (3).

On the other hand, molecular subtypes, to some extent, also indicated possibly different routes of EC development and differences in cancer microenvironmental features. Specifically, immune components in the cancer microenvironment have attracted increasing attention in recent years due to their potential roles in predicting patients’ prognosis and guiding immune checkpoint blockade therapies (4, 5). Improvements in methodologies, including single-cell and spatial transcriptomics, immune deconvolution algorithms (6) and multiplex immunofluorescence assays (7), have significantly promoted research in cancer immune microenvironment. In 2018, European researchers, for the first time, established an immune risk score based on tumor-infiltrating T cells in colon cancer tissue and validated its effectiveness in predicting recurrence in a large retrospective cohort (8). In EC, previous findings have indicated the prognostic value of immune-related gene signatures (9–11). However, most previous studies on the immune microenvironmental features of EC were only based on data of next-generation sequencing. Furthermore, extensive data regarding the association of tumor-infiltrating immune cells with patients’ molecular features are still needed to establish incorporated risk stratification systems for clinical applications.

In this study, we aimed to analyze the tumor immune microenvironmental features in different molecular subtypes of EC using multiplex immunofluorescence method, so as to provide a better understanding of the mechanisms underlying the differences in prognosis and immunotherapeutic efficacy among different EC subtypes.

## Materials and methods

### Study population and data collection

This retrospective study included 30 EC cases treated at Peking University People’s Hospital from December 2018 to November 2021. The cases were consecutively included on the condition that for each case the genetic testing and PD-L1 immunohistochemical assays were performed on fresh surgical specimens. We avoided using archived pathological specimens for the above assays so as to guarantee the accuracy of the testing results. Among all the eligible patients, 26 were primary cases and 4 were recurrent cases. All surgeries were conducted by experienced gynecologic oncologists in our center. For all early-stage primary EC cases, surgical staging was conducted, including total hysterectomy + bilateral salphingoophrectomy ± pelvic lymphnectomy ± paraaortic lymphnectomy ± omentectomy. Hysterectomy was performed through either open or laparoscopic approaches, following the routine procedures (12). Pelvic washing was collected during surgeries and sent for cytology testing. For advanced-stage primary EC cases, cytoreductive surgery was conducted. Postoperative adjuvant therapies, including chemotherapies and radiotherapies, were delivered based on patients’ clinicopathological risk factors. For recurrent cases, surgery was performed in an individualized manner, and postoperative chemotherapies were delivered. All pathological reviews were finished in the Department of Pathology of Peking University People’s Hospital by two independent gynecologic pathologists. When disconcordance occurred in pathological diagnosis, the case was sent to the expert committee of the Department of Pathology for a final diagnosis. Patients’ clinical data, including age, height and weight at diagnosis, disease history, disease stage, and pathological information were retrieved from the electronic medical record system of the hospital. The staging was determined according to the International Federation of Gynecology and Obstetrics (FIGO) 2009 staging system (13). Histopathological classification was performed according to the World Health Organization (WHO) 2014 classification system (14). The grading of tumors was in accordance with the FIGO criteria (15). The study was approved by the Institutional Review Board of Peking University People’s Hospital (2022PHB097-001).

### Genetic testing

(1) Sample processing and DNA extraction: Formalin-fixed paraffin-embedded (FFPE) tissue sections were stained with hematoxylin and eosin (H&E) to evaluate tumor cell content. Samples with a tumor content of ≥ 20% were used for subsequent analyses. After deparaffinization, the samples were incubated together with lysis buffer and proteinase K at 56°C overnight until completely digested. Then the lysate was incubated at 80°C for 4 hours to reverse formaldehyde crosslinks. Genomic DNA was extracted with the ReliaPrepTM FFPE gDNA Miniprep System (Promega) and then quantified using the QubitTM dsDNA HS Assay Kit (Thermo Fisher Scientific). For each sample subject to the following steps, a final concentration of DNA ≥ 0.6 ng/μL was needed, and the total content of DNA was required to be ≥ 30 ng.

(2) Library preparation and targeted capture: DNA extracts were fragmented by an S220 focused ultrasonicator (Covaris). Then, we prepared libraries using the KAPA Hyper Prep Kit (KAPA Biosystems). For each library, the concentration and size distribution of DNA fragments were quantified using a Qubit 3.0 fluorometer (Thermo Fisher Scientific) and a LabChip GX Touch HT Analyzer (PerkinElmer) respectively. The DNA concentration was approximately 50-80 ng/μL, and the length of the DNA fragments was approximately 390 bp. The library was then subjected to hybridization with probes targeting 733 cancer-related genes. The probe baits were individually synthesized 5′ biotinylated 120 bp DNA oligonucleotides (IDT). Repetitive elements were filtered out from intronic baits according to the annotation by UCSC Genome RepeatMasker (16). The xGen^®^ Hybridization and Wash Kit (IDT) was used for hybridization enrichment. The concentration and fragment size distribution of the final library were quantified with a Qubit 3.0 fluorometer (Thermo Fisher Scientific) and a LabChip GX Touch HT Analyzer (PerkinElmer) respectively.

(3) DNA sequencing and data processing: The final libraries were loaded onto a NovaSeq 6000 platform (Illumina) for paired-end sequencing with a mean sequencing depth of 800-1000×. Raw sequencing data were then mapped to the reference human genome hg19 with the Burrows−Wheeler Aligner (v0.7.12) (17). PCR duplicate reads were removed with Picard (v1.130), and sequence metrics were collected with SAMtools (v1.1.19). Single nucleotide variants and indels were then analyzed. Variants were filtered by their unique supporting read depth, strand bias and base quality based on the method in a previous study (18). Single-nucleotide polymorphism (SNPs) were annotated by ANNOVAR against the databases dbSNP (v138), 1000Genome and ESP6500 (population frequency > 0.015). Finally, only missense, silent, nonsense, frameshift and non-frameshift indel mutations were kept.

(4) Determination of microsatellite status: In this study, microsatellite status was determined according to the previously described algorithm (19). We examined 100 microsatellite loci, and the top 30 loci with the best coverage were used for microsatellite-instability (MSI) score calculation. The model for determining the stability of each locus is as follows:


$$P\left(X=n_{i}\right)=C_{N_{i}}^{n_{i}}p_{i}^{n_{i}}{\left(1-p_{i}\right)}^{N_{i}-n_{i}}$$

In the model, i is the locus being examined, p_i_ is the cumulative percentage at the cut-point repeat length of the microsatellite-stable (MSS) subtype, n_i_ represents the number of unstable reads, and N_i_ represents the total number of reads for that locus. A locus was considered unstable if P (X ≥ n_i_) ≤ 0.001. An MSI score was defined as the percentage of unstable loci. Any sample with an MSI score of ≥ 0.4 was classified as MSI high (MSI-H).

(5) Calculation of tumor mutation burden (TMB): TMB was defined as the number of somatic mutations per 1 Mb in examined coding regions, excluding driver mutations. Tumor somatic mutations include missense, silent, nonsense, frameshift and non-frameshift indel mutations in coding regions.

### Molecular classification of EC cases

**Figure 1:**
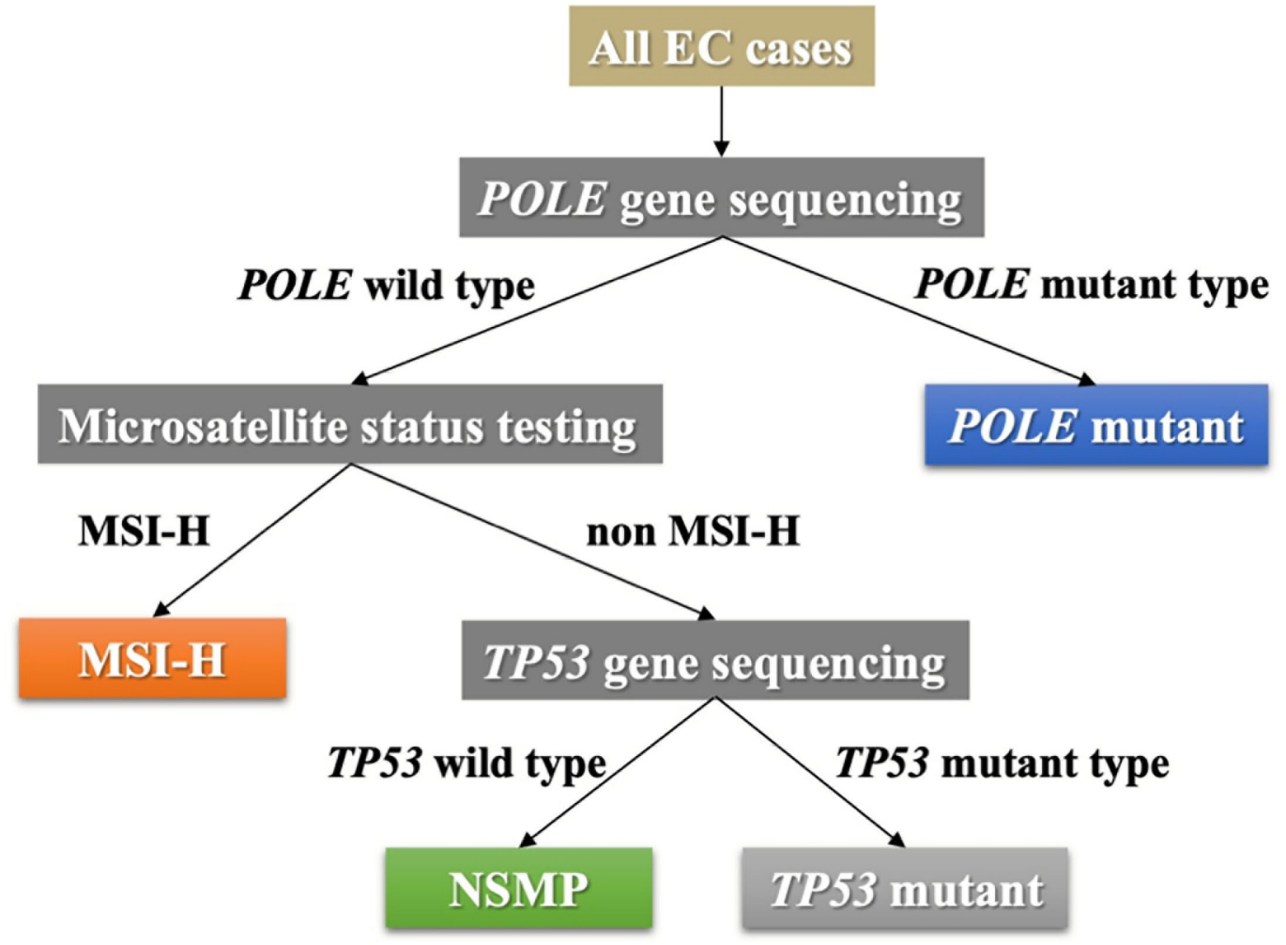
The pipeline of molecular subtyping of EC. EC, endometrial cancer; MSI-H, microsatellite instability high; NSMP, no specific molecular profile.

The molecular subtype of each EC case was determined according to *POLE* gene status, microsatellite status, and *TP53* gene status. The pipeline for subtyping was designed in accordance with the transPORTEC classification system (20), as shown in **Figure 1**. Four molecular subtypes (*POLE* mutant, MSI-H, no specific molecular profile [NSMP], and *TP53* mutant) were identified accordingly.

### PD-L1 immunohistochemical testing

PD-L1 expression levels of each sample were tested with a PD-L1 IHC 22C3 pharmDx assay (Agilent Technologies). The expression level of PD-L1 was quantified using tumor proportion score (TPS), which is defined as the percentage of viable tumor cells with partial or complete membrane PD-L1 staining at any intensity. In this study, positive PD-L1 expression was defined as TPS ≥ 1% (21).

### Testing of tumor infiltrating immune cells

For each sample, infiltrating immune cells were examined using multiplex immunofluorescence staining, which was conducted with the Akoya OPAL Polaris 7-Color Automation IHC kit (NEL871001KT), following the manufacturer’s guide. FFPE tissue slides were first deparaffinized in a BOND RX system (Leica Biosystems), which was followed by epitope retrieval. Then, the slides were incubated with primary antibodies in two panels. In panel 1, the primary antibodies against CD163 (Abcam, ab182422, 1:500), CD8 (Abcam, ab178089, 1:200), CD68 (Abcam, ab213363, 1:1000), PD-1 (CST, D4W2J, 86163S, 1:200), PD-L1 (CST, E1L3N, 13684S, 1:400) and pan-CK (Abcam, ab7753, 1:100) were added sequentially. In panel 2, the primary antibodies against CD20 (DAKO, L26, IR604, 1:1), CD3 (DAKO, A0452, 1:1), CD56 (Abcam, ab75813, 1:1000), CD4 (Abcam, ab133616, 1:100), FOXP3 (Abcam, ab20034, 1:100) and pan-CK (Abcam, ab7753, 1:100) were added sequentially. After incubating with each primary antibody, the samples were incubated with secondary antibodies and the corresponding reactive Opal fluorophores (see **Table S1** for details). Nuclei acids were stained with DAPI. Slides bound with primary and secondary antibodies but without fluorophores were used as negative controls. The tissue slides were scanned by the Vectra Polaris Quantitative Pathology Imaging System (Akoya Biosciences) at 20 nm wavelength intervals from 440 nm to 780 nm, with a fixed exposure time and an absolute magnification of ×200. All scans were then superimposed to obtain a single image for each slide. The cellular phenotype identification was performed as described previously (22). Briefly, the images were imported into inForm v.2.4.8 (Akoya Biosciences) for image analysis, and deconvolution was performed based on a multinomial logistic regression model, according to the manufacturer’s guideline. The files generated were then imported into HALO^®^ (Indica Labs) for cellular quantifications. For each case, the entire tissue section was used for analysis. Tumor parenchyma and mesenchyme were differentiated according to pan-CK staining, and were also verified by pathological review of H&E stained slides. The percentage of a certain immune cell type was defined as the percentage of positively stained cells among all nucleated cells. We calculated the fraction of CD8^+^ T cells, regulatory T cells (T_reg_ cells, CD3^+^ CD4^+^ FOXP3^+^), M1 macrophages (CD68^+^ CD163^-^), M2 macrophages (CD68^+^ CD163^+^), CD56 dimly stained natural killer (CD56^dim^ NK) cells, PD-L1^+^ CD68^+^ cells and CD8^+^ PD-1^+^ cells in the tumor parenchyma and mesenchyme accordingly.

### Statistical analysis

In this study, all intergroup comparisons were performed based on the data obtained from tissue sections of multiple samples in each group. For categorical variables, Fisher’s exact test was used to compare the differences among groups. For continuous variables, the normality of the data distribution was tested. Variables in accordance with normal distribution were described with the mean value and standard deviation (SD), and intergroup comparisons were conducted with one-way analysis of variance (ANOVA). Variables not in accordance with normal distribution were described with the median value and interquartile range (IQR), and the Kruskal−Wallis test was conducted to compare the differences among groups. All statistical analyses were performed using SPSS 26.0 (IBM Corporation, Armonk, NY, USA) and R 4.1.0 (https://www.r-project.org/). In all tests, two-sided *p* values were used. Statistically significant differences were considered when *p*< 0.05.

## Results

### Clinicopathological and molecular features of EC cases

In this study, 26 primary EC cases were included, including three of the *POLE* mutant subtype, six of the MSI-H subtype, eight of the NSMP subtype, and nine of the *TP53* mutant subtype (see **Figure S1** for the mutational profiles of all patients). The mean age of all patients was 62.38 years. Compared with the other three subtypes, the *TP53* mutant subtype showed numerically higher age at diagnosis (66.56 ± 10.90 y) and tended towards a larger proportion of postmenopausal patients (88.9%), although the differences were not significant. Body mass index (BMI) and disease history were similar across the four molecular subtypes. Three patients had other malignancies, including one case of the *TP53* mutant subtype with cooccurring ovarian cancer, and two cases of the MSI-H subtype with a history of colon cancer. One of the MSI-H cases was later diagnosed as Lynch syndrome. (**Table 1**)

**Table 1:** Clinicopathological characteristics of primary EC cases^a^.

Characteristics	Total (n = 26)	*POLE* mutant ( n= 3)	MSI-H (n = 6)	NSMP (n = 8)	*TP53* mutant (n = 9)	*p* value
Age, y, mean (SD)	62.38 (8.79)	60.33 (11.02)	59.33 (8.60)	60.75 (4.20)	66.56 (10.90)	0.381^b^
BMI, kg/m^2^, mean (SD)	26.04 (4.15)	25.07 (2.88)	24.49 (1.99)	28.09 (6.45)	25.57 (2.47)	0.399^b^
Postmenopause, No. (%)						0.208
No	6 (23.1)	2 (66.7)	2 (33.3)	1 (12.5)	1 (11.1)	
Yes	20 (76.9)	1 (33.3)	4 (66.7)	7 (87.5)	8 (88.9)	
Hypertension, No. (%)						0.817
No	13 (50.0)	1 (33.3)	4 (66.7)	4 (50.0)	4 (44.4)	
Yes	13 (50.0)	2 (66.7)	2 (33.3)	4 (50.0)	5 (55.6)	
Diabetes, No. (%)						0.931
No	19 (73.1)	2 (66.7)	5 (83.3)	6 (75.0)	6 (66.7)	
Yes	7 (26.9)	1 (33.3)	1 (16.7)	2 (25.0)	3 (33.3)	
Other malignancies, No. (%)						0.278
No	23 (88.5)	3 (100.0)	4 (66.7)	8 (100.0)	8 (88.9)	
Yes	3 (11.5)	0	2 (33.3)	0	1 (11.1)	
Pathological type, No. (%)						<0.001
Endometrioid	19 (73.1)	3 (100.0)	6 (100.0)	8 (100.0)	2 (22.7)	
Non-endometrioid	7 (26.9)	0	0	0	7 (77.8)	
FIGO stage, No. (%)						0.028
Early (stage I - II)	18 (69.2)	3 (100.0)	6 (100.0)	6 (75.0)	3 (33.3)	
Advanced (stage III - IV)	8 (30.8)	0	0	2 (25.0)	6 (66.7)	
Grade^c^, No. (%)						0.415
Low (grade 1-2)	17 (65.4)	2 (66.7)	5 (83.3)	8 (100.0)	2 (100.0)	
High (grade 3)	2 (7.7)	1 (33.3)	1 (16.7)	0	0	
Depth of myometrial invasion, No. (%)						0.269
<50%	14 (53.8)	3 (100.0)	3 (50.0)	5 (62.5)	3 (33.3)	
≥50%	12 (46.2)	0	3 (50.0)	3 (37.5)	6 (66.7)	
Cervical stromal invasion, No. (%)						1.000
No	22 (84.6)	3 (100.0)	5 (83.3)	7 (87.5)	7 (77.8)	
Yes	4 (15.4)	0	1 (16.7)	1 (12.5)	2 (22.2)	
Lymphovascular space invasion, No. (%)						0.175
No	17 (68.0)	3 (100.0)	5 (83.3)	6 (75.0)	3 (37.5)	
Yes	8 (32.0)	0	1 (16.7)	2 (25.0)	5 (62.5)	
Adnexal involvement, No. (%)						0.223
No	20 (76.9)	3 (100.0)	6 (100.0)	6 (75.0)	5 (55.6)	
Yes	6 (23.1)	0	0	2 (25.0)	4 (44.4)	
Lymph node metastasis^d^, No. (%)						0.178
No	20 (76.9)	3 (100.0)	6 (100.0)	7 (87.5)	4 (57.1)	
Yes	4 (15.4)	0	0	1 (12.5)	3 (42.9)	
Peritoneal cytology, No. (%)						0.283
Negative	22 (91.7)	2 (66.7)	6 (100.0)	8 (100.0)	6 (85.7)	
Positive	2 (8.3)	1 (33.3)	0	0	1 (14.3)	
Surgical approach, No. (%)						0.024
Open	9 (34.6)	1 (33.3)	2 (33.3)	0	6 (66.7)	
Minimally invasive	17 (65.4)	2 (66.7)	4 (66.7)	8 (100.0)	3 (33.3)	
Postoperative chemotherapy, No. (%)						0.005
No	10 (38.5)	3 (100.0)	3 (50.0)	4 (50.0)	0	
Yes	16 (61.5)	0	3 (50.0)	4 (50.0)	9 (100.0)	
Postoperative radiotherapy, No. (%)						0.619
No	17 (65.4)	3 (100.0)	3 (50.0)	5 (62.5)	6 (66.7)	
Yes	9 (34.6)	0	3 (50.0)	3 (37.5)	3 (33.3)	

^a^ For some characteristics, the number of cases did not sum up to the heading totals due to missing data. ^b^ One-way ANOVA test, all others were by Fisher’s exact test. ^c^ Grade was only determined and calculated in endometrioid EC. ^d^ Only cases receiving lymph node resections were included in the calculation. MSI-H, microsatellite instability high; NSMP, no specific molecular profile; BMI, body mass index; FIGO, International Federation of Gynecology and Obstetrics; EC, endometrial cancer.

We found significant differences in pathological types across different molecular subtypes (*p*< 0.001). All seven patients with non-endometrioid EC had *TP53* mutations, and of these seven patients, one had carcinosarcoma and six had uterine serous carcinoma. The percentage of advanced-stage cases in the *TP53* mutant subtype was the highest among all the molecular subtypes (66.7%, *p* = 0.028). Deep myometrial invasion, cervical stromal invasion, lymphovascular space invasion, adnexal involvement, and lymph node metastasis were more common in the *TP53* mutant subtype than in other subtypes, but the differences were not statistically significant, possibly due to the relatively small sample size. For all primary cases, no patient received neoadjuvant therapies. The proportion of patients receiving open surgery (*p* = 0.024) and postoperative chemotherapies (*p* = 0.005) was significantly higher in the *TP53* mutant subtype than in the other subtypes (**Table 1**)

We also included four recurrent EC cases in this study, with two of the NSMP subtype and two of the *TP53* mutant subtype. Detailed clinicopathological and molecular genetic information on the recurrent cases are shown in **Table 2** and **Figure S1**.

**Table 2:** Clinicopathological and molecular features of recurrent EC cases.

Characteristics	Patient A	Patient B	Patient C	Patient D
Age at recurrence, y	65	55	67	68
Time of recurrence	Second time	First time	First time	First time
Disease-free interval, months	42	15	14	7
Site of recurrence	Abdominal wall	Chest wall	Ilium	Paraaortic lymph node
Pathological type of recurrent tumor	Endometrioid	Endometrioid	Endometrioid	Clear cell
Grade^a^	Grade 3	Grade 3	Grade 3	–
Molecular subytpe	*TP53* mutant	NSMP	NSMP	*TP53* mutant
Chemotherapy before sampling	Yes	Yes	Yes	Yes
Radiotherapy before sampling	Yes	No	No	No
Targeted therapy before sampling	No	No	No	No

a Grade was only determined in endometrioid EC. EC, endometrial cancer; NSMP, no specific molecular profile.

### TMB levels of different molecular subtypes of EC

**Figure 2:**
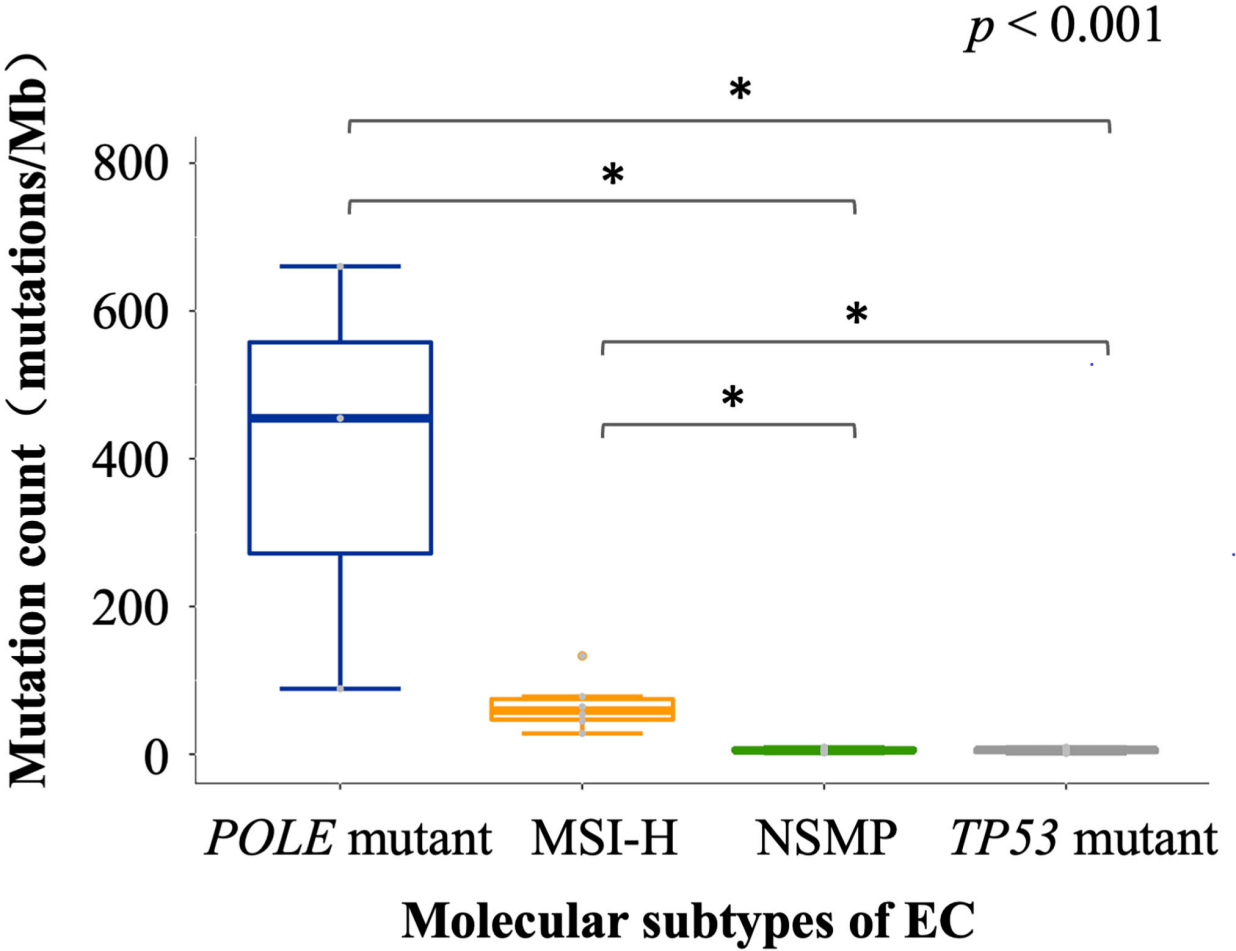
TMB of different molecular subtypes of EC. The *p* value of Kruskal-Wallis test for overall comparison is given, and significant levels in pairwise comparisons are shown in the figure. In the comparisons, n = 3 for *POLE* mutant, 6 for MSI-H, 10 for NSMP, 11 for *TP53* mutant. The dot above the boxplot indicates an outlier. MSI-H, microsatellite instability high; NSMP, no specific molecular profile; EC, endometrial cancer; TMB, tumor mutation burden. *p < 0.05.

We analyzed the tumor immune microenvironmental features of the 30 EC cases, including TMB, infiltration of antitumor-related immune cells and negatively regulatory immune cells, and the expression of immune checkpoint molecules. Consistent with TCGA data (1), patients with *POLE* mutations showed the highest level of TMB, followed by the MSI-H subtype, NSMP, and *TP53* mutant subtypes (**Figure 2**). Recent studies have indicated that TMB is highly associated with tumor-infiltrating immune cells, PD-L1 expression, and patients’ prognosis in both endometrial cancer and other cancer types (23–25). Additionally, considering the relatively small sample size of the *POLE* mutant and MSI-H subtypes, we combined the two subtypes into the TMB high (TMB-H) subtype in the following analysis.

### Infiltration of immune cell subsets in different molecular subtypes of EC

**Figure 3:**
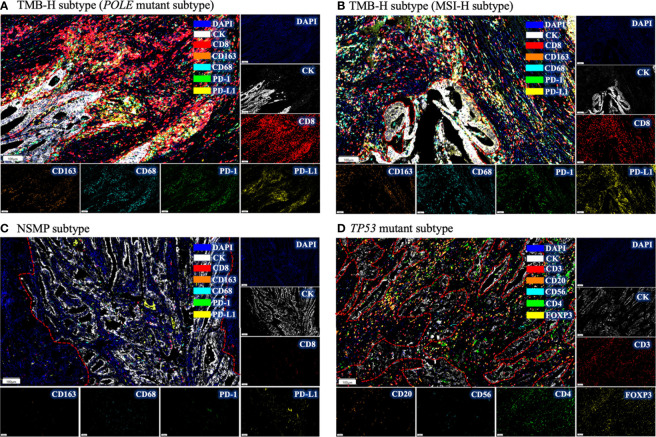
Immune infiltration in different molecular subtypes of EC by multiplex immunofluorescence. **(A)** The immune infiltrations in EC of *POLE* mutant subtype. Intense red fluorescence indicates large amount of CD8^+^ cell infiltration. **(B)** The immune infiltration in EC of MSI-H subtype. **(C)** The immune infiltration in NSMP subtype. Few fluorescence signals could be observed, indicating absence of immune infiltration. **(D)** The immune infiltration in *TP53* mutant subtype. Intense yellow fluorescence indicates the infiltration of FOXP3^+^ cells. For each subtype, a representative filed was selected, and the major tumor regions are outlined. TMB-H, high tumor mutation burden; MSI-H, microsatellite instability high; NSMP, no specific molecular profile; EC, endometrial cancer.

**Figure 4:**
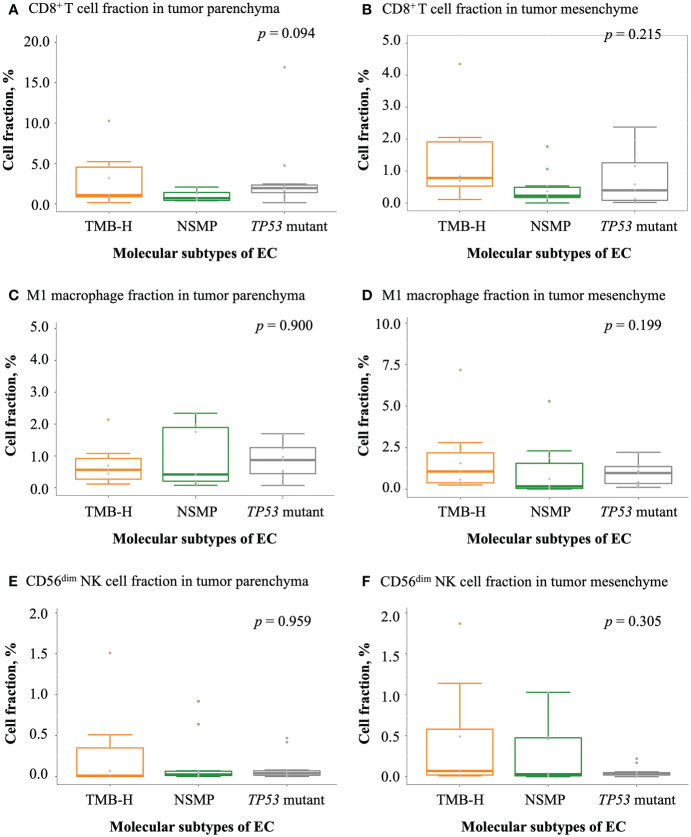
Infiltration of antitumor-related immune cells in EC. **(A, B)** CD8^+^ T cell fractions in tumor parenchyma and mesenchyme. **(C, D)** M1 macrophage fractions in tumor parenchyma and mesenchyme. **(E, F)** CD56^dim^ NK cell fractions in tumor parenchyma and mesenchyme. For **(A-F)**, the *p* values of Kruskal-Wallis test for overall comparisons are given. In all panels, n = 9 for TMB-H, 10 for NSMP, 11 for *TP53* mutant. The dots above the boxplots indicate outliers. TMB-H, high tumor mutation burden; NSMP, no specific molecular profile; EC, endometrial cancer; CD56^dim^ NK cell, CD56 dimly stained natural killer cell.

We examined tumor-infiltrating immune cells in the three molecular subtypes using multiplex immunofluorescence assays (**Figure 3** and **Figure S2**). The fractions of CD8^+^ T cells in both the tumor parenchyma and the tumor mesenchyme were higher in the TMB-H and *TP53* mutant subtypes than in the NSMP subtype, although the differences were not statistically significant (*p* = 0.094 for tumor parenchyma, *p* = 0.215 for tumor mesenchyme). The infiltration of M1 macrophages and CD56^dim^ NK cells did not differ significantly among the three subtypes. (**Figure 4** and **Table S2**)

**Figure 5:**
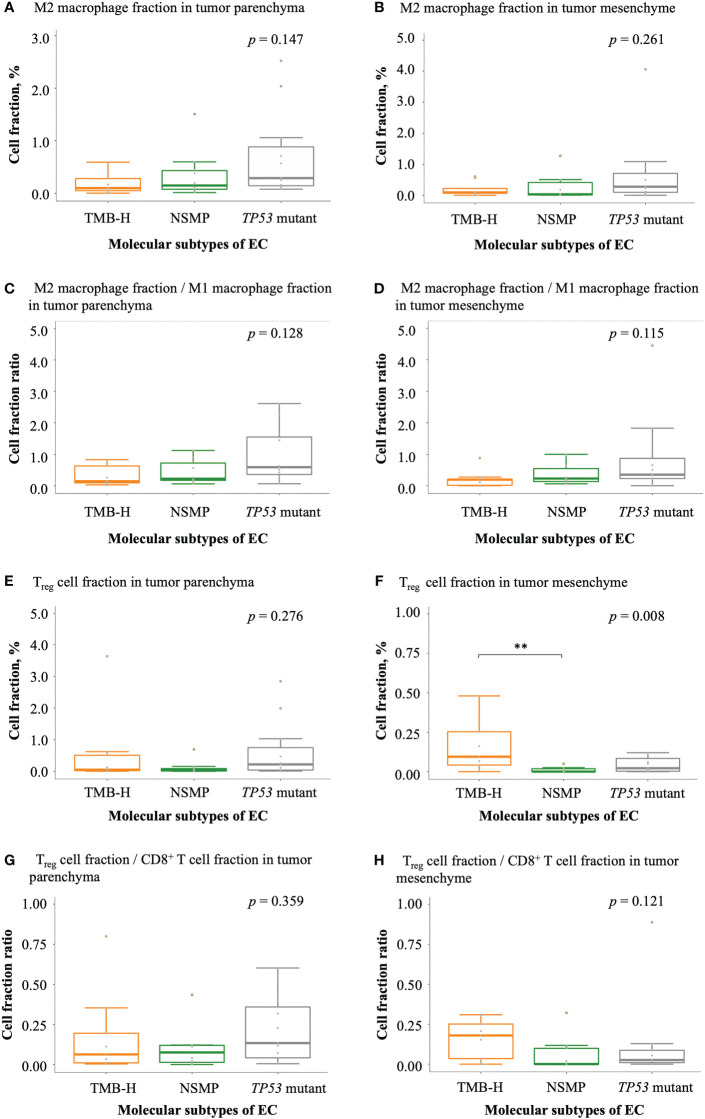
Infiltration of negatively regulatory immune cells in EC. **(A, B)** M2 macrophage fractions in tumor parenchyma and mesenchyme. **(C, D)** The ratio of M2 macrophage fractions to M1 macrophage fractions in tumor parenchyma and mesenchyme. **(E, F)** T_reg_ cell fractions in tumor parenchyma and mesenchyme. **(G, H)** The ratio of T_reg_ cell fractions to CD8^+^ T cell fractions in tumor parenchyma and mesenchyme. The *p* values of Kruskal-Wallis test for overall comparisons are given, and significant levels in pairwise comparisons are shown in the figure. In all panels, n = 9 for TMB-H, 10 for NSMP, 11 for *TP53* mutant. The dots above the boxplots indicate outliers. TMB-H, high tumor mutation burden; NSMP, no specific molecular profile; EC, endometrial cancer; T_reg_ cell, regulatory T cell. **p < 0.01.

With regard to negatively regulatory immune cells, a trend of a higher degree of infiltration of M2 macrophages was observed in tumors of the *TP53* mutant subtype comparing with tumors of the other two subtypes (**Figures 5A, B**). Similar trends were also observed in the ratios of M2 macrophage fractions to M1 macrophage fractions (**Figures 5C, D**). The percentage of T_reg_ cells and the ratio of T_reg_ cell fractions to CD8^+^ T cell fractions in the tumor parenchyma were the highest in the *TP53* mutant subtype among the three molecular subtypes, but the differences were not significant. Interestingly, we noticed that the fraction of T_reg_ cells in the tumor mesenchyme was the highest in the TMB-H subtype (*p* = 0.008), but the difference in the ratio of T_reg_ cell fractions to CD8^+^ T cell fractions in the tumor mesenchyme was not significant across the three subtypes (**Figures 5E–H** and **Table S3**).

### Expression of immune checkpoint molecules in different molecular subtypes of EC

**Figure 6:**
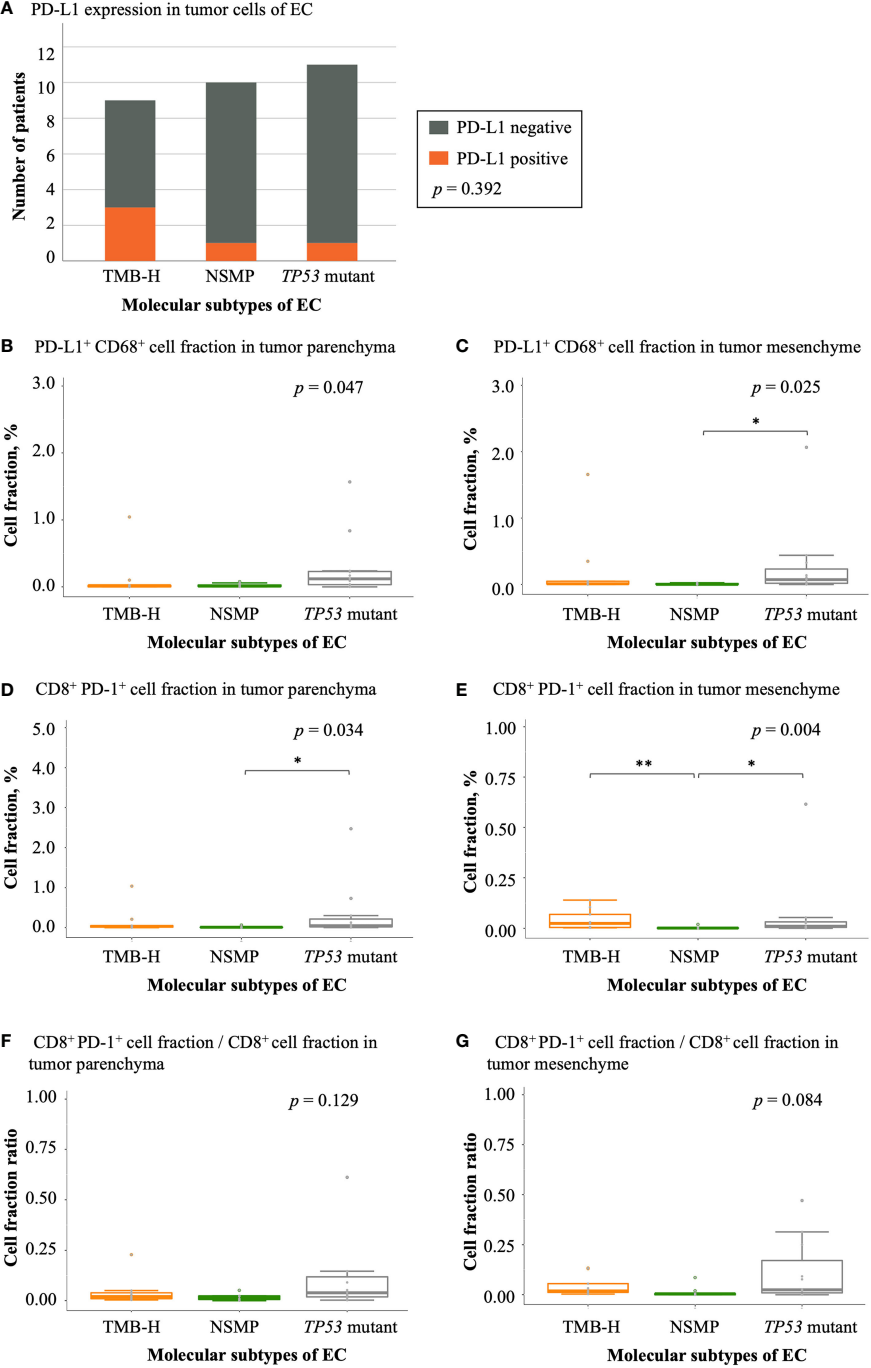
Expression of immune checkpoint molecules in EC. **(A)** PD-L1 expression in different EC molecular subtypes. The *p* value of Fisher’s exact test for overall comparison is given. **(B, C)** PD-L1^+^ CD68^+^ cell fractions in tumor parenchyma and mesenchyme. **(D, E)** CD8^+^ PD-1^+^ cell fractions in tumor parenchyma and mesenchyme. **(F, G)** The ratio of CD8^+^ PD-1^+^ cell fractions to CD8^+^ T cell fractions in tumor parenchyma and mesenchyme. For **(B-G)**, the *p* values of Kruskal-Wallis test for overall comparisons are given, and significant levels in pairwise comparisons are shown in the figure. In all panels, n = 9 for TMB-H, 10 for NSMP, 11 for *TP53* mutant. The dots above the boxplots indicate outliers. TMB-H, high tumor mutation burden; NSMP, no specific molecular profile; EC, endometrial cancer. *p < 0.05, **p < 0.01.

We also analyzed the expression of PD-L1 and PD-1 in tumor samples of the three molecular subtypes. The TMB-H subtype showed the highest rate of positive PD-L1 expression in tumor cells (33.3%), although the difference was not significant (**Figure 6A** and **Table S4**). The *TP53* mutant subtype had the largest fraction of PD-L1^+^ CD68^+^ macrophages in both the tumor parenchyma and mesenchyme (*p* = 0.047 and 0.025, respectively). In tumor parenchymal regions, CD8^+^ PD-1^+^ T cell infiltrations were the highest in the *TP53* mutant subtype among all three molecular subtypes (*p* = 0.034). In the tumor mesenchyme, the fraction of CD8^+^ PD-1^+^ T cells was higher in the TMB-H and *TP53* mutant subtypes compared with that in the NSMP subtype (*p* = 0.004), yet the proportion of CD8^+^ PD-1^+^ T cells in all CD8^+^ T cells was the highest in the *TP53* mutant subtype among all three molecular subtypes, although not significant enough (*p* = 0.084). (**Figure 6** and **Table S4**)

## Discussion

EC is one of the most common gynecologic malignancies worldwide (26). Personalized treatment strategies for EC are essential both for better precision care and for reducing treatment-related health economic burdens. With the emerging trend of applying immunotherapies in EC treatment, and the increasing evidence indicating the potential role of immunological features in predicting treatment responses (27–29), understanding tumor immune microenvironmental features and the associations with molecular features of cancer is necessary to guide immunotherapy design and predict patients’ prognosis. In this study, we compared the clinicopathological features of different molecular subtypes of primary EC. Additionally we systemically analyzed the association of EC molecular subtypes with tumor immune microenvironmental features using experimental approaches. The information provided here could be informative for the design of relevant basic and clinical studies in the future.

Our data revealed that *TP53* mutant EC was associated with non-endometrioid histology, advanced stage, and multiple negative prognostic factors, indicating compromised survival outcomes. These results were in accordance with previous studies (20, 30–33). The following analysis of the tumor immune microenvironment could, to some extent, provide explanations for this. Indeed, in recent years, studies based on cancer genomics have indicated that immunological subtypes could be used to predict patient prognosis (5). There are also available models for predicting clinical response to immune checkpoint blockade therapies (34, 35). Nevertheless, current models are still not sufficiently accurate, and further explorations are warranted.

Previous studies have indicated that increased infiltration of intratumoral CD8^+^ T cells is associated with a better prognosis in different cancer types (36–38), and a recent study based on multiplex immunofluorescent assays further supported the prognostic value of tumor infiltrating T cells in early-stage endometrial cancer (39). In this study, we also observed relatively high percentages of CD8^+^ T cells in samples of TMB-H EC, which is believed to have a favorable survival outcome (23). However, studies have shown that the functional status of CD8^+^ T cells changes with tumor progression, and different stages of dysfunctional T cells, characterized by the expression of specific immune checkpoint molecules, are thought to be associated with distinct response rates to immune checkpoint inhibitors (40). In this study, we noticed that *TP53* mutant EC showed abundant infiltration of CD8^+^ PD-1^+^ T cells in the tumor parenchyma and a high proportion of CD8^+^ T cells with PD-1 expression in the tumor mesenchyme, indicating unfavorable clinical outcomes of *TP53* mutant EC.

Innate immune cells, including macrophages and NK cells, could also modulate the tumor immune microenvironment and regulate antitumor responses. Macrophages can be further divided into M1 macrophages and M2 macrophages based on cell surface markers and functions. M1 macrophages mainly show antitumoral functions, while M2 macrophages promote tumor progression *via* stimulating tumor cell proliferation, angiogenesis, and epithelial-mesenchymal transitions (41). According to our data, a trend of higher fractions of M2 macrophages in *TP53* mutant EC relative to the other molecular subtypes was observed. This could possibly help explain the higher rate of advanced-stage diseases in this subtype as a result of tumor progression. Besides, NK cells could also be divided into two types, CD56^dim^ and CD56^bright^ (CD56 brightly stained NK cells). CD56^bright^ NK cells are mainly responsible for secreting cytokines, while CD56^dim^ NK cells show more potent cytotoxic effects (42). One recent study indicated that low NK cell infiltration in the tumor was associated with worse survival (43). However, based on our data, the fraction of CD56^dim^ NK cells was not significantly different among different EC molecular subtypes, possibly due to the limited sample size.

T_reg_ cells are another vital cell type with prognostic significance. Evidence has shown that high T_reg_ cell infiltration level is associated with compromised survival and hyperprogression of disease following immune checkpoint blockade therapy (44, 45). But interestingly enough, in this study, a higher proportion of T_reg_ cells was observed in the tumor mesenchyme of the TMB-H subtype, instead of the *TP53* mutant subtype, which are commonly thought to have poor survival. According to previous studies, T_reg_ cells responsible for modulating immune responses typically express the same transcription factors, which are also expressed in the cells that they regulate; moreover, in antitumor immune responses, T_reg_ cells could be triggered by the same chemotaxis molecules that also recruit CD8^+^ T cells to the tumor site (46). Therefore, the higher proportion of CD8^+^ T cells in the tumor mesenchyme in TMB-H tumors may have contributed to the higher proportion of T_reg_ cells. Furthermore, the similar ratio of T_reg_ cells to CD8^+^ T cells in the three molecular subtypes also supported the above hypothesis.

In summary, EC patients with high TMB showed abundant tumor-infiltrating CD8^+^ T cells and relatively high levels of PD-L1 expression in tumor cells, which is consistent with data from previous studies on *POLE* mutant and MSI-H EC (47–49). The above indicates that there are strong antitumor immune responses in TMB-H tumors and that this subtype is potentially suitable for immune checkpoint blockade therapies. In the NSMP subtype, the TMB, the proportions of multiple tumor-infiltrating immune cells and the expression levels of immune checkpoint molecules were low, indicating a lack of effective antitumor immune responses. In the *TP53* mutant subtype, the TMB level was low. However, the proportions of T_reg_ cells, M2 macrophages, PD-L1^+^ CD68^+^ macrophages and CD8^+^ PD-1^+^ T cells were relatively high, indicating a strong immune suppressive microenvironment in this molecular subtype. Based on the immune microenvironmental features analyzed above, we summarize the immune phenotype of the three molecular subtypes as normal immune response, absence of immune infiltration, and suppressed immune response.

The distribution pattern of immune cells in the tumor tissue could also indicate differences in their functions. Recently, Keren et al. (50) proposed a model for describing immune infiltrative patterns in the tumor: compartmentalized pattern, which suggested that tumor cells and immune cells form relatively independent regions; cold pattern, which indicated low levels of tumor infiltrating immune cells; and mixed pattern, which implied the highly mixed distribution of tumor and immune cells. Among these, patients with the compartmentalized pattern showed better survival than those with the mixed pattern (50). In this study, we also analyzed the distribution of immune cells in the tumor parenchyma and mesenchyme. Our study showed that CD8^+^ T cells were distributed in both tumor parenchymal and mesenchymal regions in TMB-H EC. The fraction of CD8^+^ T cells in the tumor mesenchyme was the highest in the TMB-H subtype, which means that a part of T cells could form a relatively isolated region adjacent to the tumor cells. Among the three molecular subtypes, the NSMP subtype displayed the lowest degree of CD8^+^ T cell infiltration. In the *TP53* mutant subtype, CD8^+^ T cells were mostly distributed in tumor parenchyma, with a relatively low median level of infiltration in tumor mesenchyme. The features of CD8^+^ T cell distribution in the three molecular subtypes, to some extent, resemble that of the model mentioned above (50). Besides, *TP53* mutant EC typically shows the worst survival (1, 20, 30–33) across all subtypes, which is also consistent with the survival features revealed by the above model (50). Another recent study analyzed the interactions of cellular neighborhoods in colorectal cancer with distinct immune infiltrative features (51). In brief, in tumors with numerous tertiary lymphoid structures, T cell exchange between the T cell cluster and tumor invasive front could help enhance the antitumor immune response; while in tumors with diffuse inflammatory infiltration, the immune suppressive macrophage cluster showed strong contact with the tumor invasive front and inhibited effective immune responses (51). The mechanisms described above might also explain the distinct survival features in EC of different molecular subtypes and immune infiltrative patterns.

Notably, in this study, both the TMB-H subtype and *TP53* mutant subtype showed high levels of immune checkpoint molecule expression. However, in the TMB-H subtype, PD-L1 was mainly expressed in tumor cells, while in the *TP53* mutant subtype, there were high levels of PD-L1 expression in macrophages and high levels of PD-1 expression in T cells. These results indicate that the cellular distribution of immune checkpoint molecules could also provide information on the immune response status of the patient and differences in patients’ responses to immune checkpoint blockade therapies. Further studies with larger sample sizes are needed to confirm the findings.

In this study, we quantitively analyzed tumor immune microenvironmental features in different EC molecular subtypes and provided information about the spatial distributions of multiple immune cell types in the tumor tissue. A key strength is that we adopted experimental methods for *in situ* visualization of the cells, which showed direct evidence for tumor immune infiltrations. In this regard, this study could provide a vital supplement to previous studies based on bulk tissue sequencing and computational deconvolution. By systemically analyzing the TMB, tumor infiltrating immune cells and immune checkpoint molecules, we summarized the immunophenotypes of different EC molecular subtypes, thus providing clues for understanding their distinct survival features and treatment responses. However, there are also some limitations. First, the sample size of the study was relatively small, which limited the statistical power in some analyses. Studies with larger sample sizes are needed to further validate our findings. Second, since most cases in this study were treated recently and follow-ups are on-going, survival information is still lacking. Long-term follow-up is necessary to analyze the associations of tumor immune microenvironmental features with patients’ recurrence, survival and responses to multiple treatment modalities. Finally, as an explorative study, the panel we used for testing tumor-infiltrating immune cells included multiple cellular markers. Further explorations are needed to refine the testing strategies and develop clinically feasible panels for better practical applications.

Currently, with the deep clinical influence of TCGA molecular subtyping and its surrogate methods (1, 20, 31), the diagnosis and treatment of EC are becoming increasingly more comprehensive and individualized. Molecular markers provide vital information and rationality for applying targeted drugs and immune checkpoint blockade therapies in specific patient groups (52). Nevertheless, heterogeneity in prognosis and treatment response could still be seen even within the same pathological and molecular subtype, which urges further refinement of the current risk stratification system (3). During this effort, barriers still exist that the cellular architectures of the tumor tissues and the biological behaviors of malignant and surrounding stromal and immune cells are far less understood in EC. As indicated above (50, 51), this information may also be highly associated with clinical outcomes. The results from this study, on the one hand, established the connection between molecular subtypes and the immune microenvironmental features of EC. On the other hand, it paved the way for further designing related studies. We encourage more efforts using multiplex imaging methods to establish a prognostic or treatment-related classification system based on immunological markers. Based on these efforts, incorporating effective immunological features into current EC patients’ risk stratification systems would be another vital step for better individualized treatment.

## Data availability statement

The raw sequence data reported in this paper have been deposited in the Genome Sequence Archive (53) in National Genomics Data Center (54), China National Center for Bioinformation / Beijing Institute of Genomics, Chinese Academy of Sciences (GSA-Human: HRA003458) that are publicly accessible at https://ngdc.cncb.ac.cn/gsa-human. The raw data of multiplex immunofluorescence assays have been deposited in the OMIX, China National Center for Bioinformation / Beijing Institute of Genomics, Chinese Academy of Sciences (https://ngdc.cncb.ac.cn/omix: accession no. PRJCA013221).

## Ethics statement

The studies involving human participants were reviewed and approved by Institutional Review Board of Peking University People’s Hospital. The patients/participants provided their written informed consent to participate in this study. Written informed consent was obtained from the individual(s) for the publication of any potentially identifiable images or data included in this article.

## Author contributions

YD and ZW contributed to study conception and design. Development of methodology was performed by YD, DH, LC, LZ and ZW. Acquisition of data was conducted by YD, LZ, XZ, NK, LQ, LL and HL. Data analysis was performed by YD, DH and LZ. The first draft of the manuscript was written by YD, and the manuscript was reviewed and revised by LC, JW and ZW. All authors contributed to the article and approved the submitted version.

## Funding

This work was supported by the National Key Technology Research and Developmental Program of China (2022YFC2704300, 2022YFC2704303), the National Natural Science Foundation of China (81972426) and the Capital’s Funds for Health Improvement and Research (2022-2Z-4086, 2022-1-4081).

## Acknowledgments

We would like to thank Dr. Xiaochen Zhao from 3D Medicines Inc., for her help in the development of methodology and data interpretation, and Dr. Ting Bei, who is from 3D Medicines Inc., for her help in the review of this manuscript.

## Conflict of interest

Authors DH and LC were employed by 3D Medicines Inc.

The remaining authors declare that the research was conducted in the absence of any commercial or financial relationships that could be construed as a potential conflict of interest.

## Publisher’s note

All claims expressed in this article are solely those of the authors and do not necessarily represent those of their affiliated organizations, or those of the publisher, the editors and the reviewers. Any product that may be evaluated in this article, or claim that may be made by its manufacturer, is not guaranteed or endorsed by the publisher.
