# Supplementary material for: Tumor immune microenvironment in endometrial cancer of different molecular subtypes: evidence from a retrospective observational study
Source: Front Immunol. 2022 Nov 24;13:1035616. doi: 10.3389/fimmu.2022.1035616 (PMC9756131; doi:10.3389/fimmu.2022.1035616)
Supplement: Supplementary file 1 [file DataSheet_1.docx]

Supplementary Material

# Supplementary Tables

**Table S1.** Primary antibodies and fluorophores used in multiplex immunofluorescence assays

| **Panels** | **Target** | **Source** | **Dilution** | **Opal fluorophores** | **Color** |
| --- | --- | --- | --- | --- | --- |
| Panel 1 | CD8 | Abcam, ab178089 | 1:200 | Opal 690 | Red |
|  | CD68 | Abcam, ab213363 | 1:1000 | Opal 480 | Cyan |
|  | CD163 | Abcam, ab182422 | 1:500 | Opal 620 | Magenta |
|  | PD-1 | CST, D4W2J, 86163S | 1:200 | Opal 520 | Green |
|  | PD-L1 | CST, E1L3N, 13684S | 1:400 | Opal 570 | Yellow |
|  | pan-CK | Abcam, ab7753 | 1:100 | Opal 780 | White |
| Panel 2 | CD3 | DAKO, A0452 | 1:1 | Opal 690 | Red |
|  | CD56 | Abcam, ab75813 | 1:1000 | Opal 480 | Cyan |
|  | CD20 | DAKO, L26, IR604 | 1:1 | Opal 620 | Magenta |
|  | CD4 | Abcam, ab133616 | 1:100 | Opal 520 | Green |
|  | FOXP3 | Abcam, ab20034 | 1:100 | Opal 570 | Yellow |
|  | pan-CK | Abcam, ab7753 | 1:100 | Opal 780 | White |

**Table S2.** Antitumor-related immune cells in EC of different molecular subtypes

| **Immune cell type** | **TMB-H**  **(n=9)** | **NSMP**  **(n=10)** | ***TP53* mutant**  **(n=11)** | ***p* value** |
| --- | --- | --- | --- | --- |
| CD8^+^ T cell fraction in tumor parenchyma, %, median (IQR) | 1.08 (0.87-4.55) | 0.71 (0.44-1.41) | 1.94 (1.39-2.33) | 0.094 |
| CD8^+^ T cell fraction in tumor mesenchyme, %, median (IQR) | 0.78 (0.53-1.91) | 0.23 (0.17-0.49) | 0.40 (0.08-1.26) | 0.215 |
| M1 macrophage fraction in tumor parenchyma, %, median (IQR) | 0.56 (0.27-0.92) | 0.42 (0.21-1.89) | 0.87 (0.44-1.26) | 0.900 |
| M1 macrophage fraction in tumor mesenchyme, %, median (IQR) | 1.07 (0.38-2.19) | 0.17 (0.05-1.56) | 0.98 (0.35-1.37) | 0.199 |
| CD56^dim^ NK cell fraction in tumor parenchyma, %, median (IQR) | 0.01 (0.00-0.35) | 0.03 (0.01-0.07) | 0.04 (0.02-0.07) | 0.959 |
| CD56^dim^ NK cell fraction in tumor mesenchyme, %, median (IQR) | 0.07 (0.02-0.58) | 0.03 (0.01-0.48) | 0.03 (0.02-0.05) | 0.305 |

Abbreviations: EC, endometrial cancer; TMB-H, high tumor mutation burden; NSMP, no specific molecular profile; IQR, interquartile range; CD56^dim^ NK cell, CD56 dimly stained natural killer cell.

**Table S3.** Negatively regulatory immune cells in EC of different molecular subtypes

| **Immune cell type** | **TMB-H**  **(n=9)** | **NSMP**  **(n=10)** | ***TP53* mutant**  **(n=11)** | ***p* value** |
| --- | --- | --- | --- | --- |
| M2 macrophage fraction in tumor parenchyma, %, median (IQR) | 0.10 (0.05-0.28) | 0.15 (0.08-0.43) | 0.29 (0.15-0.88) | 0.147 |
| M2 macrophage fraction in tumor mesenchyme, %, median (IQR) | 0.09 (0.07-0.22) | 0.32 (0.01-0.41) | 0.28 (0.10-0.71) | 0.261 |
| M2 macrophage/M1 macrophage in tumor parenchyma, median (IQR) | 0.14 (0.09-0.63) | 0.22 (0.17-0.72) | 0.59 (0.36-1.55) | 0.128 |
| M2 macrophage/M1 macrophage in tumor mesenchyme, median (IQR) | 0.19 (0.01-0.20) | 0.23 (0.14-0.55) | 0.35 (0.23-0.87) | 0.115 |
| T_reg_ cell fraction in tumor parenchyma, %, median (IQR) | 0.05 (0.02-0.51) | 0.05 (0.01-0.09) | 0.22 (0.04-0.75) | 0.276 |
| T_reg_ cell fraction in tumor mesenchyme, %, median (IQR) | 0.10 (0.04-0.25) | 0.00 (0.00-0.02) | 0.02 (0.00-0.08) | 0.008 |
| T_reg_ cell/CD8^+^ T cell in tumor parenchyma, median (IQR) | 0.06 (0.01-0.20) | 0.08 (0.01-0.12) | 0.13 (0.04-0.36) | 0.359 |
| T_reg_ cell/CD8^+^ T cell in tumor mesenchyme, median (IQR) | 0.18 (0.03-0.25) | 0.00 (0.00-0.10) | 0.03 (0.01-0.09) | 0.121 |

Abbreviations: EC, endometrial cancer; TMB-H, high tumor mutation burden; NSMP, no specific molecular profile; IQR, interquartile range; T_reg_ cell, regulatory T cell.

**Table S4.** Expression of immune checkpoint molecules in EC

| **Immune checkpoint molecule** | **TMB-H**  **(n=9)** | **NSMP**  **(n=10)** | ***TP53* mutant**  **(n=11)** | ***p* value** |
| --- | --- | --- | --- | --- |
| PD-L1 expression in tumor cells |  |  |  | 0.392^a^ |
| Negative, No. (%) | 6 (66.7) | 9 (90.0) | 10 (90.9) |  |
| Positive, No. (%) | 3 (33.3) | 1 (10.0) | 1 (9.1) |  |
| PD-L1^+^ CD68^+^ cell fraction in tumor parenchyma, %, median (IQR) | 0.02 (0.00-0.02) | 0.01 (0.01-0.03) | 0.12 (0.03-0.23) | 0.047 |
| PD-L1^+^ CD68^+^ cell fraction in tumor mesenchyme, %, median (IQR) | 0.01 (0.01-0.05) | 0.00 (0.00-0.01) | 0.07 (0.02-0.23) | 0.025 |
| CD8^+^ PD-1^+^ cell fraction in tumor parenchyma, %, median (IQR) | 0.03 (0.01-0.05) | 0.01 (0.01-0.01) | 0.05 (0.02-0.22) | 0.034 |
| CD8^+^ PD-1^+^ cell fraction in tumor parenchyma, %, median (IQR) | 0.02 (0.00-0.07) | 0.00 (0.00-0.00) | 0.01 (0.00-0.03) | 0.004 |
| CD8^+^ PD-1^+^ cell/CD8^+^ T cell in tumor parenchyma, median (IQR) | 0.02 (0.01-0.04) | 0.02 (0.01-0.02) | 0.04 (0.02-0.12) | 0.129 |
| CD8^+^ PD-1^+^ cell/CD8^+^ T cell in tumor parenchyma, median (IQR) | 0.02 (0.01-0.06) | 0.00 (0.00-0.01) | 0.02 (0.01-0.17) | 0.084 |

^a^ Fisher’s exact test, all others were by Kruskal−Wallis test. Abbreviations: EC, endometrial cancer; TMB-H, high tumor mutation burden; NSMP, no specific molecular profile; IQR, interquartile range.

# Supplementary Figures

#



**Figure S1.** The mutational profiles of eligible cases. Abbreviations: TMB, tumor mutation burden; MSI-H, microsatellite instability high; NSMP, no specific molecular profile.


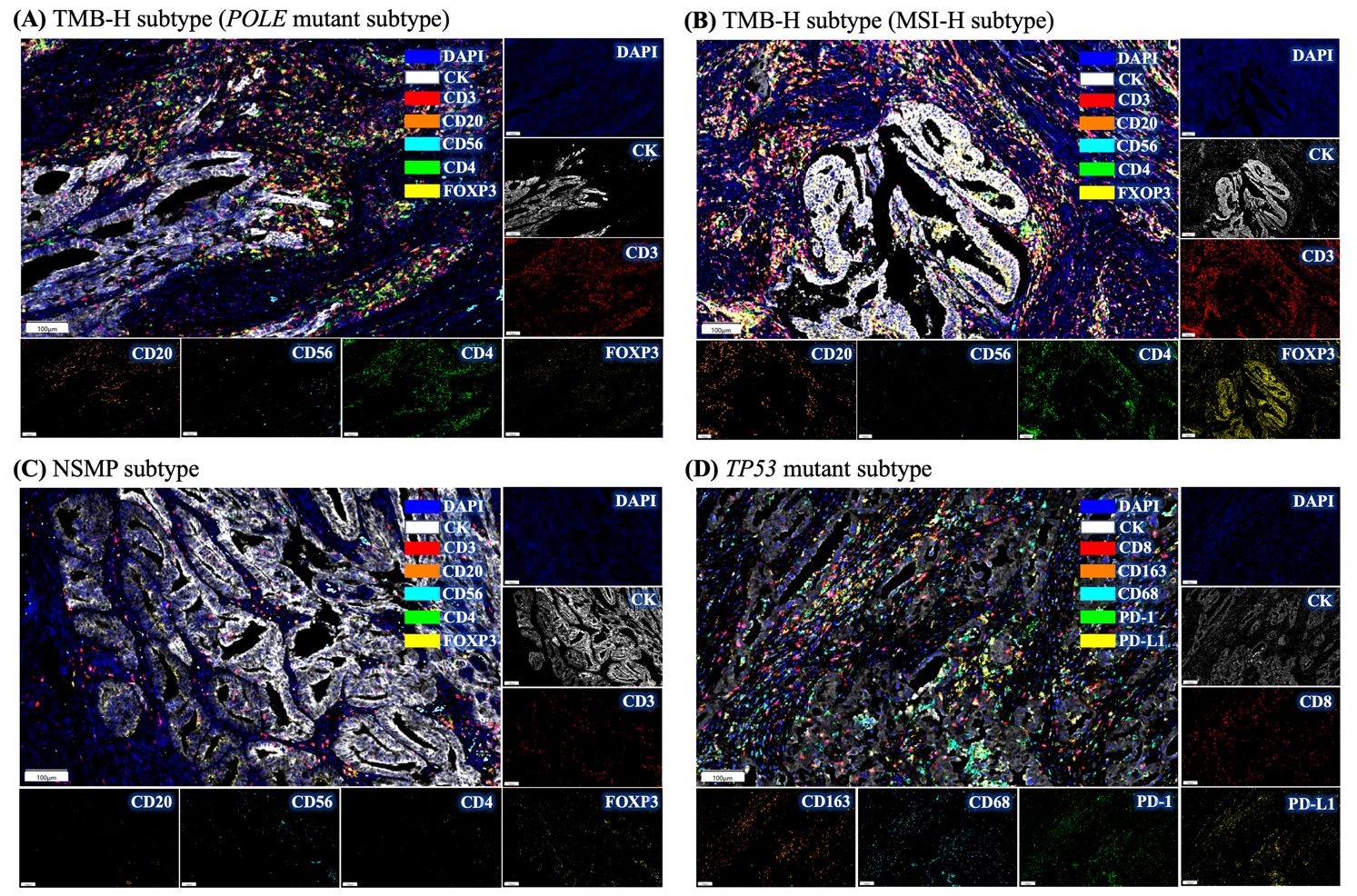


**Figure S2.** The multiplex immunofluorescent images of different molecular subtypes of EC. For each subtype, the figures are in complement to the panels shown in Figure 2. Abbreviations: EC, endometrial cancer; TMB-H, high tumor mutation burden; MSI-H, microsatellite instability high; NSMP, no specific molecular profile.
